# Cross-Domain Deep Transfer Learning Framework for Intrusion Detection in Data-Constrained and Resource-Limited IoT Environments

**DOI:** 10.3390/s26175441

**Published:** 2026-08-28

**Authors:** Imran Mohammed, Imad Mahgoub

**Affiliations:** Department of Electrical Engineering and Computer Science (EECS), Florida Atlantic University, Boca Raton, FL 33431, USA; imohamme@fau.edu

**Keywords:** internet of things, intrusion detection, data-constrained environments, edge devices, deep transfer learning

## Abstract

The rapid expansion of the Internet of Things (IoT) has led to the widespread deployment of interconnected smart devices and wireless sensor systems in critical applications, significantly increasing the attack surface for cyber threats. Deep learning (DL)-based intrusion detection systems (IDSs) have demonstrated considerable potential for enhancing IoT security through their ability to automatically learn complex attack patterns and detect anomalous behavior. However, the effectiveness of these approaches often depends on the availability of large volumes of labeled training data, which are difficult to obtain in many IoT environments due to device heterogeneity, evolving attack patterns, privacy constraints, and the limited availability of domain-specific intrusion datasets. Consequently, conventional DL-based IDSs often exhibit reduced performance under data-constrained conditions. To address these challenges, this paper proposes a deep transfer learning (DTL)-based intrusion detection framework for data-constrained and resource-limited IoT environments. The proposed approach leverages knowledge acquired from large-scale computer network intrusion datasets by pre-training deep neural network (DNN) models on source-domain data and subsequently fine-tuning them using smaller IoT intrusion datasets. In addition, pruning and quantization techniques are incorporated to reduce model complexity and enable efficient deployment on resource-constrained IoT edge devices. Experimental results demonstrate that the proposed DTL framework outperforms conventional DL-based IDS models, achieving improvements in accuracy, recall, F1-score, and area under the receiver operating characteristic curve (AUC). Furthermore, the compressed models achieve substantial reductions in model size and improved inference latency. These findings demonstrate the effectiveness of cross-domain knowledge transfer for addressing intrusion detection in data-constrained IoT environments.

## 1. Introduction

The Internet of Things is a network of sensors, actuators, and devices—“things”—that are connected to the internet. They have hardware and software that enable them to exchange data with each other and with people and systems. These objects range from everyday consumer items like smart thermostats, wearable fitness trackers, and connected home appliances to industrial machinery, medical devices, and even vehicles. Through embedded intelligence and connectivity, these devices can monitor their environment, perform actions, gather and transmit valuable data, and respond to commands or automate processes [1].

Recent advancements in embedded systems, wireless communications and edge computing accelerated the deployment of IoT systems across numerous application domains, including healthcare, industrial automation, transportation systems, agriculture, smart cities, environmental monitoring and military systems [2]. This has enabled intelligent sensing, automation, and real-time decision-making across these domains. These systems continuously generate large volumes of heterogeneous data that can be leveraged to develop intelligent analytics and predictive models.

This interconnectedness also brings challenges, particularly concerning security and privacy because many of these devices have limited computer power and are often deployed with weak security protections. Some of the IoT security challenges involve weak authentication, insecure communication, malware attacks, poor update mechanisms, resource limitations, and device management complexity [3]. These factors make them highly vulnerable to cyberattacks.

A network intrusion or attack happens when an actor who is not authorized to access a digital network or devices on the network but does so with the intention to destabilize the system, steal, modify or delete data, or install malicious software. Improper data handling can lead to privacy violations and identity theft. Attackers can easily gain unauthorized access to devices and compromised IoT devices can become part of botnets used for cyberattacks. For example, the Mirai Botnet Attack infected thousands of IoT devices and launched massive DDoS attacks.

Security of IoT devices must be considered throughout the entire device lifecycle, and they must be secured against any type of cyberattack. One of the methods to mitigate cyberattacks is by monitoring and analyzing network traffic to detect intrusions and attacks. A system that does that is known as an intrusion detection system (IDS) [3].

IDSs are critical for monitoring and protecting IoT networks from unauthorized access and malicious activities. They are designed to monitor network traffic, analyze data patterns, logs, and events to detect malicious activity and security threats. Implementing effective IDSs in IoT environments is crucial for maintaining security and integrity. As the deployment of IoT continues to grow, the development of advance IDS solutions tailored to the unique characteristics of IoT are essential for safeguarding them.

Traditional security methods have developed various responses to cyberattacks such as blocking an attack, using honeypots to lure attackers and gather information about them. Machine learning and deep learning techniques have become fundamental components of modern IDSs due to their ability to extract meaningful insights from data and detect cyberattacks [4,5,6,7,8,9,10]. ML- and DL-based IDSs require large amounts of training data to achieve acceptable performance [11,12]. However, there is a lack of sufficient high-quality training data [13].

In real-world IoT environments, data scarcity can arise for several reasons. Newly deployed IoT infrastructures typically lack historical operational data during the initial deployment phase. Additionally, many IoT applications operate under strict privacy and security constraints that prevent centralized data collection and sharing. For example, healthcare IoT systems and wearable devices often contain sensitive personal information protected by regulatory frameworks [13].

Resource limitations further exacerbate the problem. Edge devices commonly possess constrained computational capabilities, limited storage capacity, and restricted energy resources, making continuous data collection and transmission impractical [14]. In remote or harsh environments, including underwater monitoring systems, precision agriculture, and wildlife tracking networks, intermittent connectivity and unreliable communication channels may lead to incomplete or missing data streams. Moreover, the heterogeneous nature of IoT devices introduces inconsistencies in sensing modalities, sampling rates, and data quality, thereby reducing the availability of usable labeled datasets.

Traditional supervised learning approaches generally require large-scale labeled datasets to achieve acceptable performance. Consequently, such approaches may not be effective in sparse-data IoT scenarios. To address these limitations, recent research has explored alternative learning paradigms that improve data efficiency and enable robust model adaptation under limited-data conditions.

In this paper we propose a deep transfer learning framework for intrusion detection in data-constrained IoT environments by leveraging knowledge from both IoT and computer networks domain. In the proposed framework, a data-constrained IoT environment refers to a deployment scenario in which only a limited amount of labeled IoT intrusion data is available for model training due to high cost and effort associated with security-data annotation, privacy and data-sharing constraints, device and network heterogeneity, or the emergence of new attack types. Our framework combines deep learning with the power of transfer learning to significantly improve the detection of cyberattacks and enables robust model adaptation under limited-data conditions.

In addition to cross-domain knowledge transfer, the proposed framework incorporates a model compression stage to improve its suitability for deployment in resource-constrained IoT environments. Following the transfer learning and fine-tuning processes, the trained model is compressed to reduce complexity and memory requirements while maintaining intrusion detection performance. By integrating model compression with deep transfer learning, the framework not only addresses data scarcity through knowledge transfer from computer network and IoT domains but also enables efficient deployment on edge devices with limited resources.

Key contributions:

The main contributions of this work are as follows:

- We propose a novel deep transfer learning framework for IoT intrusion detection that integrates knowledge from both IoT and traditional computer network domains.
- We develop a cross-domain learning methodology that enables the effective utilization of computer network intrusion data to address data scarcity challenges in IoT security applications.
- We incorporate model compression techniques into the proposed framework to reduce complexity and memory requirements, enabling efficient deployment on resource-constrained IoT edge devices.
- We conduct extensive experiments and analysis to evaluate the proposed framework, demonstrating its ability to improve intrusion detection performance in data-constrained IoT environments.

The rest of the paper is organized as follows. In Section 2, we review the related literature on approaches to data scarcity in IoT. Section 3 gives a background on deep leaning, transfer learning and deep transfer learning. Section 4 describes our datasets and methodology. Section 5 describes our experiments and in Section 6 we discuss our results. In Section 7 we draw conclusions and talk about the next steps.

## 2. Related Work

Recent research efforts have increasingly focused on addressing data scarcity and limited-label challenges in IoT systems. Various studies have explored the integration of advanced machine learning paradigms to improve model robustness and adaptability under constrained data availability.

### 2.1. Transfer Learning

Transfer learning has been extensively explored as a solution to data scarcity in IoT applications. Existing studies have successfully applied transfer learning to anomaly detection, intrusion detection, industrial fault diagnosis, wastewater treatment systems, and energy consumption forecasting. These approaches leverage knowledge acquired from related source domains to improve learning performance when target-domain data are limited. Reported benefits include enhanced classification accuracy, improved forecasting performance, and reduced dependence on labeled datasets.

A transfer learning framework for anomaly detection in multivariate IoT traffic data, designed to address the challenge of limited labeled data, is presented in [15]. The study demonstrates that the proposed model can effectively identify anomalies in entirely unlabeled datasets and outperform existing techniques in terms of accuracy. In [16], the authors introduce a transfer learning-based intrusion detection framework tailored for 5G IoT environments. Reference [17] investigates transfer learning as a strategy for addressing the challenges associated with learning from scarce data. A key application is demonstrated in the context of a Wastewater Treatment Plant (WWTP), where transfer learning enhances the performance of machine learning algorithms despite limited training data. In [18], a transfer learning approach for motor-bearing fault diagnosis under data-scarce conditions is proposed. The study highlights its suitability for industrial applications where labeled data are limited. An adaptive transfer learning framework for HVAC power consumption forecasting is presented in [19]. The framework leverages diverse source domains and a deep learning architecture incorporating attention mechanisms to improve generalization, accuracy, and robustness in data-scarce scenarios.

Despite these advances, transfer learning methods remain vulnerable to domain mismatch and negative transfer, particularly in IoT environments where source and target data distributions differ significantly.

### 2.2. Federated Learning

Federated learning has emerged as a promising framework for enabling distributed model training while preserving data privacy. In IoT environments, federated learning has been applied to large-model adaptation on resource-constrained devices, sensor fault classification, and renewable energy forecasting. Several studies have combined federated learning with complementary techniques such as transfer learning, knowledge distillation, and data augmentation to address challenges associated with limited local datasets.

In [20], the authors propose Federated Fine-tuning Adaptive Aggregation via Knowledge Distillation (FedFKD), a method designed to improve the training of large models on resource-constrained IoT devices. The approach addresses data scarcity and catastrophic forgetting by dynamically adjusting aggregation weights and combining information from both recent and historical data. In [21], a sensor fault classification method that integrates federated learning (FL) and transfer learning is presented, enabling effective classification across homogeneous sensor nodes using both labeled and unlabeled data. A federated learning framework for renewable energy forecasting is proposed in [22], employing a Long Short-Term Memory (LSTM) network enhanced with GAN-based data augmentation. The approach addresses the challenge of limited local data and demonstrates improved forecasting accuracy and training convergence compared with conventional methods that do not incorporate data augmentation.

While these approaches improve model performance and privacy preservation, federated learning systems continue to face challenges related to communication overhead, non-IID data distributions, device heterogeneity, and energy constraints.

### 2.3. Few-Shot and Meta-Learning

Few-shot learning and meta-learning techniques have demonstrated significant potential for addressing limited-data challenges in IoT applications. Prior research has applied these methods to intrusion detection, anomaly detection, time-series forecasting, and fault diagnosis tasks. By leveraging prior knowledge acquired from related tasks, these approaches enable rapid adaptation to new environments using only a small number of labeled samples. In addition, the integration of meta-learning with federated learning has shown promise in improving performance under highly constrained data conditions.

In [23], the authors evaluate few-shot learning classifiers for detecting previously unseen IoT network intrusions using limited labeled data. The study also examines the effectiveness of probability averaging in improving detection stability while addressing challenges such as benign-attack ambiguity and cross-device attack correlations. Reference [24] presents a bilevel optimization framework for automated time-series forecasting (TSF) under limited historical data conditions. By leveraging knowledge acquired from previously observed data, the framework enhances the learning process and demonstrates effectiveness in data-scarce scenarios. In [25], an intrusion detection system based on few-shot learning is proposed for IoT security applications. The method employs a multistage attention Siamese network to identify network traffic anomalies using minimal labeled data, thereby addressing the challenge of scarce anomaly samples. A collaborative learning framework that integrates meta-learning with federated learning for fault diagnosis under data-scarce conditions is presented in [26].

### 2.4. Synthetic Data Generation

Synthetic data generation has gained increasing attention as a means of mitigating data scarcity in IoT systems. Recent studies have employed generative AI, GANs, CTGANs, and other data augmentation techniques across applications including trajectory prediction, remote sensing classification, smart grid stability prediction, construction cost estimation, and predictive maintenance cybersecurity. These methods enhance model training by augmenting limited datasets, addressing class imbalance, and generating representative samples for rare events.

In [27], the authors present a generative AI-based framework for augmenting trajectory prediction datasets in edge-computing environments. The study demonstrates that synthetic trajectories generated by large AI models can improve prediction accuracy to levels comparable with models trained on real-world datasets. In [28], the authors propose EffBaGAN, a generative adversarial network designed for remote sensing classification in data-scarce scenarios. The model employs a balancing architecture to address class imbalance while reducing network complexity. Reference [29] utilizes a GAN-based framework for smart grid stability prediction, generating out-of-distribution (OOD) samples from stable data to enhance anomaly detection capabilities. In [30], conditional tabular generative adversarial networks (CTGANs) are employed for data augmentation in green building construction cost estimation, addressing issues related to data scarcity and class imbalance. In [31], the authors address cybersecurity challenges in predictive maintenance systems by proposing a novel methodology that leverages generative AI for data augmentation and enhanced threat detection.

Although synthetic data generation has demonstrated substantial benefits, challenges remain in ensuring the realism, reliability, diversity, and domain consistency of the generated data.

### 2.5. Data Protection and Privacy

Protecting sensitive IoT data remains a fundamental challenge due to the distributed nature of edge computing and the large volumes of personal and operational information processed by connected devices. Recent studies emphasize privacy-preserving AI techniques, including federated learning, secure model deployment, encryption, differential privacy, and confidential computing, to reduce the exposure of sensitive information during model training and inference [3].

Federated learning (FL) provides one potential approach for protecting raw IoT data by allowing models to be trained locally and sharing model updates rather than transferring raw traffic to a centralized server. However, federated learning does not inherently guarantee privacy, and model updates may still expose information through inference, reconstruction, poisoning, or other attacks [20,22]. Consequently, privacy-preserving mechanisms such as differential privacy, secure aggregation, homomorphic encryption, and secure multi-party computation may be required when sensitive IoT data are used for collaborative model training. These mechanisms introduce additional computational and communication overhead, which must be carefully considered in resource-constrained IoT environments.

### 2.6. Adversarial Attacks Against AI-Based IDS

Despite their effectiveness, deep learning models remain susceptible to adversarial attacks designed to manipulate model predictions through carefully crafted perturbations. Such attacks include evasion attacks, data poisoning, model extraction, prompt injection, and adversarial examples that exploit vulnerabilities during both training and inference. These threats are particularly concerning in cybersecurity applications because successful adversarial manipulation may cause malicious traffic to be incorrectly classified as benign or may generate misleading explanations for security analysts. Developing robust intrusion detection systems therefore requires adversarial training, input validation, uncertainty estimation, model monitoring, and continual adaptation to evolving attack strategies [31,32].

### 2.7. Multimedia Data Security

The increasing integration of cameras, microphones, smart displays, medical imaging systems, autonomous devices, and other multimedia sensors into IoT environments has expanded the scope of IoT security beyond conventional network and telemetry data. Multimedia data such as images, audio, and video may contain highly sensitive information about individuals, physical environments, locations, and operational activities. Consequently, compromising multimedia data can result in privacy violations, unauthorized surveillance, manipulation of digital evidence, or disruption of safety-critical IoT services. Recent research on intelligent multimedia forensics emphasizes the need to address security threats across both the multimedia data and the deep learning models used to analyze these data [33].

Multimedia data face several security and privacy threats, including unauthorized access, interception, manipulation, data poisoning, deepfake generation, adversarial perturbations, and inference attacks. Unlike conventional network-flow data, multimedia information contains rich semantic and contextual content, meaning that disclosure of an apparently benign image, video frame, or audio recording may expose sensitive information.

### 2.8. Relationship Between Data Security Mechanisms and Intrusion Detection Models

Intrusion detection systems represent one component of a comprehensive cybersecurity strategy and should be considered alongside complementary data security mechanisms. While traditional security mechanisms—including encryption, authentication, access control, secure communication protocols, digital signatures, and key management—are designed to prevent unauthorized access and protect the confidentiality, integrity, and availability of data, they cannot completely eliminate cyber threats [11]. Sophisticated attacks may exploit software vulnerabilities, compromised credentials, insider threats, misconfigurations, or previously unknown vulnerabilities to circumvent preventive security controls. Consequently, intrusion detection models provide an essential second layer of defense by continuously monitoring network activity, identifying anomalous behavior, and generating alerts when malicious activity is suspected [12].

The relationship between data security mechanisms and intrusion detection is particularly important in IoT environments. IoT devices often possess limited computational resources, heterogeneous hardware platforms, and diverse communication protocols, making it difficult to implement comprehensive security protections on every device [1,14]. Although encryption and authentication protect data during storage and transmission, they do not identify compromised devices, detect lateral movement, recognize command-and-control communications, or reveal abnormal behavioral patterns within encrypted network traffic. Deep learning-based intrusion detection systems address these limitations by learning normal and malicious traffic behaviors from network metadata and communication patterns rather than relying solely on packet payload inspection [12].

### 2.9. Proposed Framework

While transfer learning, federated learning, few-shot learning, and synthetic data generation have demonstrated effectiveness in mitigating data scarcity, existing studies have primarily focused on specific tasks and application domains. In the context of intrusion detection, most transfer learning approaches rely on datasets originating from a single domain, typically IoT networks. Also, most of the approaches do not support deployment on resource-constrained IoT edge devices. Addressing these challenges is essential for developing robust and scalable intrusion detection systems capable of operating effectively in both data-scarce and resource-limited IoT environments.

Motivated by these limitations, this work proposes a deep transfer learning framework for intrusion detection that leverages knowledge from both IoT and traditional computer network domains. By exploiting complementary information from multiple domains, the proposed approach aims to improve detection performance in data-constrained and resource-limited IoT environments. The literature review also indicates limited investigation of DTL-based intrusion detection frameworks that jointly leverage IoT and traditional computer network datasets, highlighting the novelty of the proposed framework. Table 1 presents a comparison between related works and our work.

The proposed framework focuses primarily on binary intrusion detection using network traffic, providing a foundation for future integration of privacy-preserving learning, adversarial robustness, and multimedia security analysis.

## 3. Transfer Learning Techniques

This section presents an overview of the concepts and techniques used in our experiments.

### 3.1. Transfer Learning Concepts

Machine learning techniques use data from the same domain to train, test/validate and predict. There are scenarios where data from a particular domain is difficult or expensive to gather or simply is not available. In such scenarios, data from a related domain, referred to as the source domain, is used to create learners for another domain, referred to as the target domain. This process of information and knowledge transfer is known as transfer learning. Transfer learning is a technique in machine learning where a pre-trained model, often trained on a large dataset or task, is adapted to learn a new task or make predictions on a different dataset.

The idea is that the pre-trained model has already learned general features and patterns that can be useful for the new task, so by fine-tuning the model’s weights on the new data, it can quickly adapt to the specific requirements of the new task.

Given a source domain Ds and learning task Ts, a target domain Dt and learning task Tt, transfer learning tries to improve the learning of the target predictive function fT by using the related information and knowledge from Ds and Ts [34]. The source and target domains may differ while the source and target tasks may be either related or different.

In transfer learning, negative transfer occurs when knowledge transferred from a source domain adversely affects performance on the target domain, resulting in lower target-domain performance [34]. This is a situation in which knowledge learned from a source domain is transferred to a target domain but does not provide useful information for the target task and instead degrades the target-domain learning performance.

Catastrophic forgetting, in contrast, refers to the loss or degradation of previously learned source-domain knowledge during fine-tuning on the target domain. Catastrophic forgetting can occur even when transfer learning improves target-domain performance and therefore should not be interpreted as evidence of negative transfer.

Thus, negative transfer concerns harm to target-domain performance caused by transfer, whereas catastrophic forgetting concerns loss of source-domain knowledge during adaptation.

### 3.2. Deep Transfer Learning

Deep transfer learning is a new paradigm of machine learning, that combines the advantages of deep learning and transfer learning to learn hidden transferable knowledge [35]. Given a transfer learning task defined by {Ds, Ts, Dt, Tt, fT (·)}, it is a deep transfer learning task in which fT (·) represents a non-linear function that corresponds to a deep neural network. DTL is classified into four categories based on the techniques used: network-based, instance-based, mapping-based and adversarial-based [35].

Popular deep learning architectures that are used for transfer learning are Feed-forward Neural Networks (FNNs) also known as Multilayer Perceptrons (MLPs), Convolutional Neural Networks (CNNs), Recurrent Neural Networks (RNNs) and Transformers.

### 3.3. Network-Based Deep Transfer Learning

**Figure 1:**
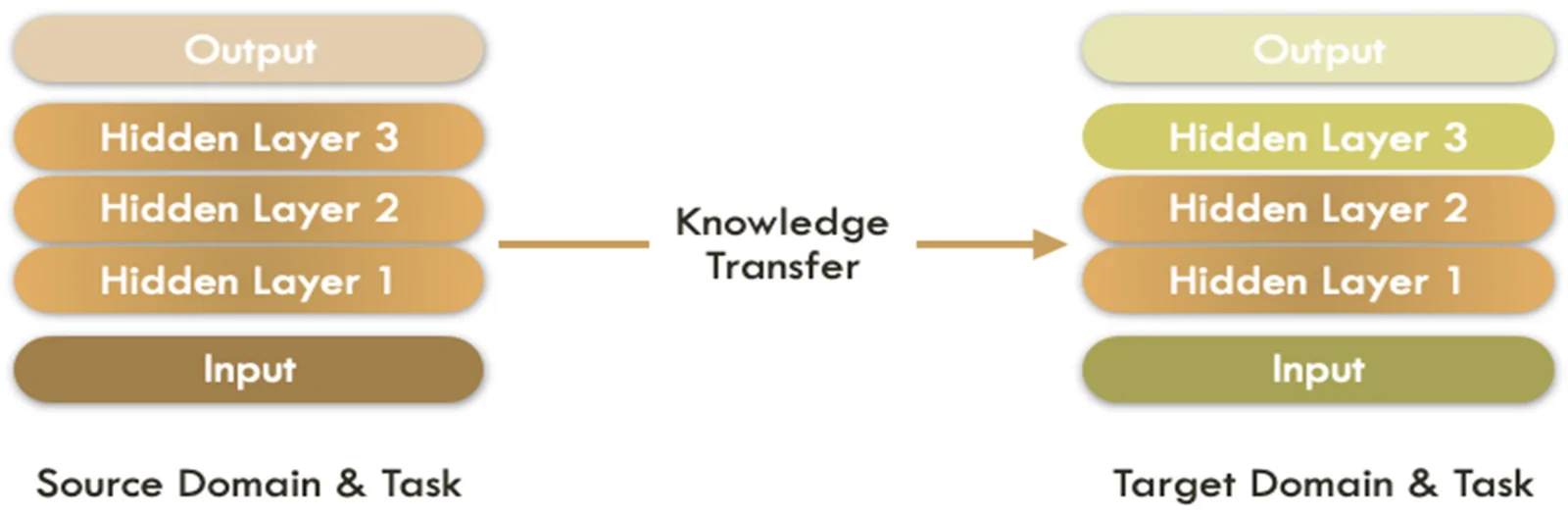
Network-based deep transfer learning.

Network-based deep transfer learning involves reusing a portion of a pre-trained network from the source domain, including its network structure and connection parameters, and incorporating it into a deep neural network used for the target domain. This approach is grounded in the idea that neural networks function similarly to the human brain’s processing mechanism, involving iterative and continuous abstraction. In this context, the initial layers of the network serve as feature extractors, and the features they extract are adaptable across different tasks [35]. Figure 1 represents the concept of network-based deep transfer learning.

In network-based deep transfer learning a deep neural network is trained on a source task, all or most of the pre-trained model’s weights are frozen, and the model is trained on the target task, while updating some or all the weights are to learn the specific features and patterns relevant to the target task. By leveraging knowledge gained from pre-training, the model can adapt more quickly to the new task.

## 4. Datasets and Methodology

### 4.1. Datasets

We used the following datasets in our experiments.

#### 4.1.1. CIC-IDS2017

This dataset contains benign and the most up-to-date common attacks, which resemble the true real-world data (PCAPs). It also includes the results of the network traffic analysis with labeled flows based on the time stamp, source, and destination IPs, source and destination ports, protocols and attack (CSV files). Also available is the extracted features definition [36].

#### 4.1.2. IOT-23

This is a dataset of network traffic from IoT devices. It has 20 malware captures executed in IoT devices, and 3 captures for benign IoT devices traffic. It was first published in January 2020, with captures ranging from 2018 to 2019. This IoT network traffic was captured in the Stratosphere Laboratory, AIC group, FEL, CTU University, Czech Republic. Its goal is to offer a large dataset of real and labeled IoT malware infections and IoT benign traffic for researchers to develop machine learning algorithms.

The IoT-23 dataset consists of twenty-three captures (called scenarios) of different IoT network traffic. These scenarios are divided into twenty network captures (pcap files) from infected IoT devices (which will have the name of the malware sample executed on each scenario) and three network captures of real IoT devices network traffic (that have the name of the devices where the traffic was captured).

The network traffic captured for the benign scenarios was obtained by capturing the network traffic of three different IoT devices: a Philips HUE smart LED lamp, an Amazon Echo home intelligent personal assistant and a Somfy smart door lock. Both malicious and benign scenarios run in a controlled network environment with unrestrained internet connection like any other real IoT device [37].

The proposed framework is formulated as a binary classification problem, where network traffic is classified as either benign or malicious. Table 2 shows the data distribution from both datasets of benign and malicious instances.

### 4.2. Methodology

One of the major drawbacks in using machine learning techniques to solve security challenges of IoT networks is lack of data [13]. Without historical data, reliable machine learning models cannot be created and trained to identify an attack.

Computer networks data and IoT data have several commonalities and differences. Similarities include volume, velocity, and variety. Differences include unique data generation patterns and diversity in data types.

- Volume: Both datasets can be large, but IoT datasets can become larger due to the continuous data generation from numerous devices.
- Velocity: IoT data is generated at a much higher speed than computer network data.
- Variety: The data in IoT comes from various sensors and devices and is heterogeneous. Computer network data may include structured and unstructured data.
- Data Generation Patterns: Computer network datasets may have static or periodic data whereas IoT devices continuously generate data in real-time.
- Diversity in Data Types: IoT datasets encompass a wide range of data types, including sensor readings, network traffic, and user interactions, which are often more heterogeneous than traditional datasets.

As can be seen from the comparison, there are more similarities than differences between IoT and computer networks data.

In our experiments, we use network activity data from computer networks and apply transfer learning techniques to classify traffic coming into an IoT network as normal or abnormal i.e., an attack. Here, the domains-computer networks and IoT are different, however the task-intrusion detection is same. Since these two domains are interconnected, transfer learning can potentially enhance the performance of a target learner. Another way to look at this transfer learning scenario is to think of the training data and the target data as belonging to distinct subdomains that are connected through a broader, overarching domain. For instance, both intrusion detection in computer networks and intrusion detection in IoT fall under the broader category of intrusion detection in a network of connected devices. The overarching domain defines the relationship between these subdomains.

#### 4.2.1. Domain Analysis and Cross-Domain Knowledge Transfer

The proposed framework assumes that computer network intrusion data and IoT network traffic share common security characteristics that enable effective cross-domain knowledge transfer. Although these two domains differ in terms of network architecture, communication protocols, and device capabilities, both exhibit similar low-level traffic behaviors associated with cyberattacks, including abnormal connection patterns, unusual packet statistics, excessive communication rates, and malicious session characteristics. These shared behavioral patterns enable a DNN pre-trained on computer network intrusion data to learn generalized feature representations that remain useful when adapted to IoT intrusion detection tasks. Table 3 summarizes the relationship between CIC-IDS2017 and IoT-23 datasets.

Although the datasets differ substantially in their traffic distributions and operating environments, both contain representative malicious behaviors that exhibit similar statistical and temporal characteristics. Consequently, the source-domain model learns general representations of malicious network activity that can be refined during fine-tuning using IoT-specific traffic. This observation provides the rationale for adopting cross-domain transfer learning in the proposed framework.

#### 4.2.2. Data-Constrained Target-Domain Setting

Although IoT-23 contains a substantially larger number of records than CIC-IDS2017, the presence of a large raw dataset does not imply that an equivalent quantity of labeled data is available in a practical IoT deployment. The objective of the proposed framework is specifically to investigate whether knowledge learned from a larger computer network intrusion dataset can improve binary intrusion detection when only a limited amount of labeled IoT data is available. Therefore, a data-constrained condition is explicitly defined as a target-domain training scenario in which only a small fraction of the available labeled IoT-23 training data is accessible to the learning algorithm.

Therefore, a controlled subset of 5000 IoT-23 records was selected for the target-domain experiments. The subset size was chosen to provide a sufficiently large number of labeled samples for stable training and evaluation while remaining small enough to represent a realistic data-constrained learning scenario.

The target subset was selected exclusively from the training portion of IoT-23 after applying the capture-disjoint partitioning strategy. Stratified sampling was used to maintain representation of the benign and malicious classes, while ensuring that the selected records were distributed across the eligible training captures rather than being dominated by a single capture scenario. The IoT-23 validation and test partitions were established independently and remained fixed throughout the experiments. Consequently, no test records were used during subset selection, preprocessing, model training, or hyperparameter optimization.

Table 4 presents the IoT-23 capture assignments used for training, validation, and testing, together with the number of benign and malicious records in each partition after cleaning and sampling. Since every malicious capture already contains a mix of Benign-labeled flows alongside the Malicious-labeled flows, the benign class for each split comes from the same captures already assigned to that split. This ensures that the capture-disjoint partitioning is preserved since both label types originate from the same capture and there is no leakage.

The choice of 5000 records should therefore be interpreted as a controlled experimental definition of a data-constrained target-domain condition, rather than as a universal threshold for limited IoT data. This fixed-size design also provides a reproducible basis for comparing the proposed DTL framework with conventional target-only learning while substantially reducing the computational burden associated with processing the complete IoT-23 dataset.

#### 4.2.3. Dataset Subset Selection and Sampling Strategy

Because CIC-IDS2017 and IoT-23 contain millions of network-flow records, using the complete datasets for every experimental configuration would impose substantial computational, memory, and storage requirements and would make repeated experiments impractical. Therefore, representative subsets of both datasets were constructed using a controlled sampling procedure. The objective of the sampling process was not to arbitrarily reduce the datasets, but to retain the statistical and semantic characteristics relevant to binary intrusion detection while ensuring that the experiments remained computationally feasible and reproducible.

For CIC-IDS2017, preprocessing was first performed to remove invalid records, duplicate flows, and attributes that were not suitable for cross-domain learning. The remaining records were mapped to the two-class problem used in this study, namely benign and malicious traffic. The source-domain subset was then selected using stratified sampling to preserve the representation of benign traffic and the available malicious traffic categories. This prevents the source model from being dominated by the most frequently occurring attack category and ensures that the pre-trained model is exposed to a diverse set of network behaviors. The selected CIC-IDS2017 subset was used for source-domain model pre-training and was kept fixed across the target-domain experiments.

For IoT-23, sampling was performed in a capture-aware manner because records originating from the same capture may exhibit strong temporal and statistical correlations. The dataset was first partitioned at the capture/scenario level into training, validation, and testing subsets. Sampling was subsequently performed only within the training partition. Thus, records from test captures were never included in the training subset. Within the eligible training captures, records were sampled according to the binary benign and malicious labels while maintaining representation across the available training captures. This approach reduces the likelihood that the selected subset is dominated by a single capture and provides a more representative approximation of the target-domain traffic distribution.

Importantly, the test partition was established before target-domain sampling and remained fixed for all experiments. The test data were not used during sampling, feature selection, normalization, hyperparameter optimization, or model selection. Consequently, increasing or decreasing the amount of target-domain training data does not change the evaluation population.

To investigate the effect of labeled target-data availability, multiple IoT-23 training subsets were subsequently generated from the eligible training partition. The subsets corresponded to 1%, 5%, 10%, and 25% of the available target-domain training records. The fractions are calculated relative to the 5000-record subset that represents the complete eligible training set used in our experiments. These percentages define the data-constrained experimental conditions used to evaluate whether transfer learning provides greater benefits when fewer labeled IoT samples are available.

This sampling strategy provides a practical compromise between computational feasibility and experimental validity. Rather than processing the complete CIC-IDS2017 and IoT-23 datasets for every experiment, the proposed procedure retains representative source-domain attack behavior, preserves target-domain capture diversity, prevents test-set contamination, and enables controlled experiments across multiple levels of labeled IoT data availability. A summary of the sampling strategy is presented in Table 5.

#### 4.2.4. Data Cleaning, Preprocessing and Feature Alignment

CIC-IDS2017 and IoT-23 were generated using different network environments and feature extraction procedures. Consequently, the raw feature representations were not assumed to be directly compatible. Before transfer learning, the features of both datasets were analyzed according to their semantic meaning, data type, and availability in the respective traffic representations. Dataset-specific identifiers and attributes without meaningful correspondence across domains were removed. Features describing common network behavior were retained and transformed into a shared representation.

The final representation contains the same number of input features for both domains, allowing the input-connected weights of the source-pre-trained network to be transferred to the target model. Where the raw feature names differ, semantic correspondence rather than name matching was used to determine whether the variables represent equivalent network characteristics.

Categorical variables were converted using a consistent numerical representation, while continuous variables were normalized using parameters derived exclusively from the appropriate training data. Preprocessing objects were never fitted using validation or test samples. During target-domain experiments, the preprocessing parameters were estimated using only the selected target training subset and subsequently applied unchanged to the validation and test data.

Because the proposed task is binary intrusion detection, the final labels were mapped to two classes: benign and malicious.

Following preprocessing, both datasets were represented using a common feature vector consisting of 29 aligned numerical features. Table 6, Table 7 and Table 8 present a list of original features, removed features, and a complete one-to-one mapping and ordering for all 29 features, for both source and target domains after cleaning and preprocessing.

To prevent data leakage, all preprocessing operations were estimated exclusively from the training partition. Numerical scaling parameters, categorical encoders, and normalization statistics were computed using only the training data and subsequently applied unchanged to the validation and testing sets.

This establishes a clear relationship between the original dataset distributions, feature-aligned representations, target-data availability, and final model evaluation. It also provides a controlled basis for determining whether the performance improvements of the proposed DTL framework arise from cross-domain knowledge transfer and are most pronounced when labeled IoT training data are genuinely limited.

## 5. Experiments

We used state-of-the-art deep learning and deep transfer learning techniques to develop and evaluate the proposed models. The implementation was developed in Python 3.12 using TensorFlow 2.16 and Keras 3, with experiments conducted using Jupyter Notebook 7.5.2 and Visual Studio Code 1.119. The experiments were performed on a macOS-based system equipped with a quad-core Intel Core i5 processor.

For our experiments, we created deep neural network models in two phases. In the first phase we created deep learning models, used the IoT dataset to train them and compared their performance against each other. In the second phase we created deep transfer learning models—created deep neural network models, used the computer networks dataset to train them, froze their weights, and fine-tuned the weights on the IoT dataset. We then compared the performance of all models against each other.

All models created are fully connected MLPs with different number of hidden layers and different number of nodes in the hidden layers. The algorithm used is backpropagation. There were two main reasons for why we chose the MLP model structure. On the one hand, it is simple, performs well, and has low computational cost; on the other hand, it can be modeled to have enough depth to capture the complexities of data and task at hand, while avoiding overfitting.

To avoid overfitting, we used regularization, a technique that constrains the values of the coefficients of the model towards zero, thereby reducing model complexity.

We used the L2 regularization, which is the sum of the squared values of the weights. All models were trained for 100 epochs using a batch size of 64 without using early stopping.

To select the best neural network architecture, twenty-five fully connected MLP models with different number of hidden layers were tested on IoT and computer network datasets. We used hyperparameter tuning to choose parameters such as the number of hidden nodes in layers, the batch size, the number of epochs, the loss function and the type of regularization. The performance of the models was compared and the model and architecture with the best metrics was chosen for further study.

In our experiments, the model that showed the best metrics was a fully connected MLP with 3 hidden layers, and hence we chose that model for further study. Table 9 and Table 10 specify the DNN and DTL architectures and hyperparameters.

The development and training phase of DTL framework consisted of two steps: pre-training and fine-tuning. In the pre-training step, we trained deep neural networks with different number of hidden layers on computer network data, calculated their accuracies and selected the network architecture and model with the best accuracy. In the fine-tuning step, we transferred the knowledge from the pre-trained model by selecting different number of layers and using them as the base of a new deep neural network.

**Figure 2:**
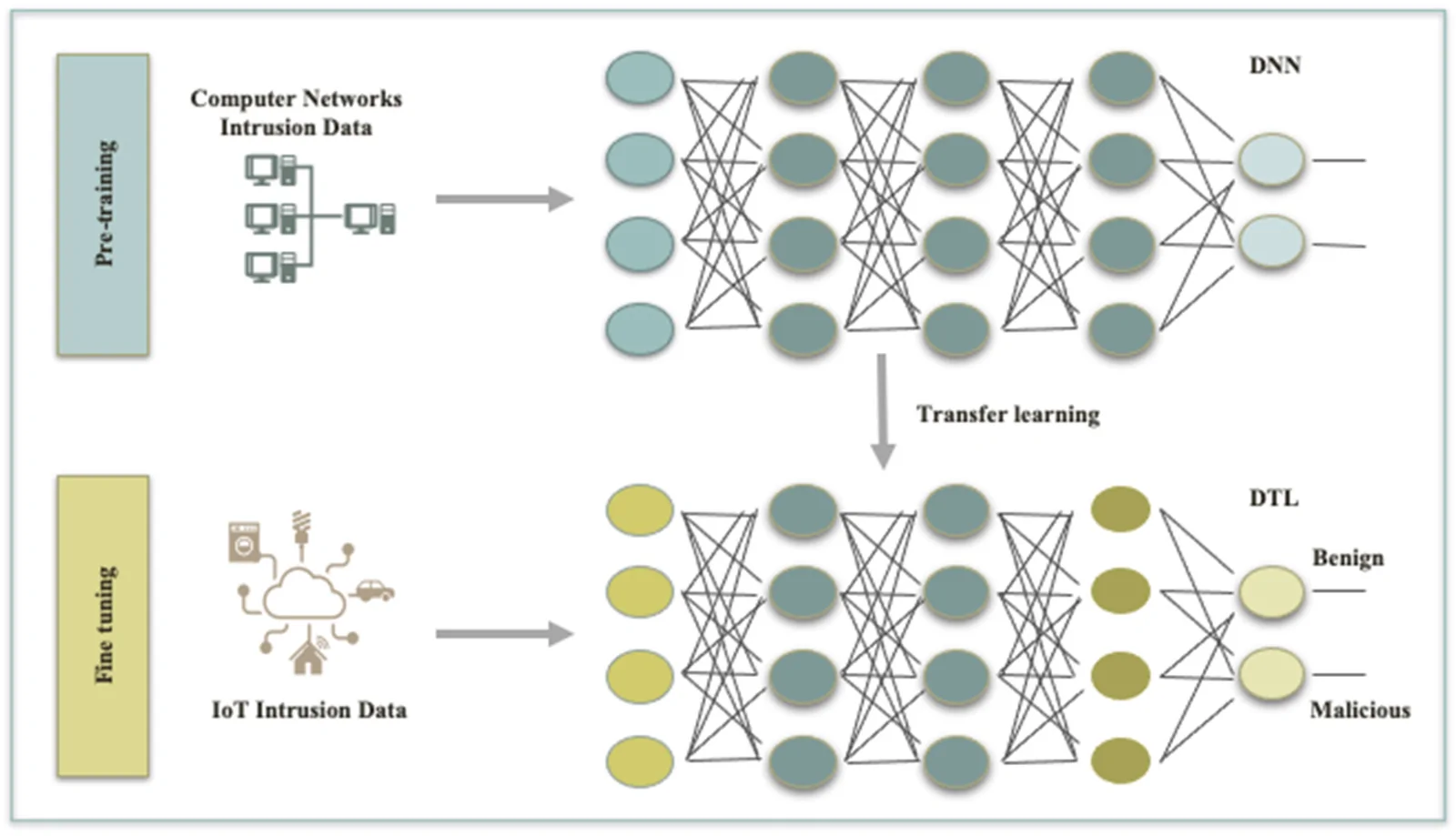
Overview of our approach.

We further added different number of hidden layers and train the models on IoT data, calculated the accuracies of the models and selected the architecture and model with the best accuracy as the final DTL model. An overview of the training and classification phases of our approach is summarized in Figure 2.

The proposed framework employs two primary learning algorithms: a conventional deep neural network (DNN) and a deep transfer learning (DTL) algorithm. The DNN is trained directly on labeled IoT traffic and serves as the target-domain baseline. The DTL algorithm first pre-trains a DNN using computer network intrusion data, then transfers selected learned layers to the IoT domain. The algorithms are presented below (Algorithms 1 and 2).
**Algorithm 1:** DNN Training**Input:**          x_t, y_t: Training samples and labels from the target domain          x_tv, y_tv: Validation samples and labels          Lmax: Maximum number of hidden layers**Output:**          M_DNN: Trained DNN**Begin**          best_score ← 0          M_DNN ← NULL          **for** i = 1 to Lmax **do**                    Construct a DNN with i hidden layers                    Initialize the model parameters                    Train the DNN using (x_t, y_t)                    Evaluate the trained model using (x_tv, y_tv)                    Compute validation performance                    **if** validation score > best_score then                              best_score ← validation score                              M_DNN ← current DNN                    **end if**          **end for**          Return M_DNN**End**

**Algorithm 2:** DTL Layer Selection and Fine-Tuning
**Input:**
          x_t, y_t: Training samples and labels from the target domain          x_tv, y_tv: Validation samples and labels          M_S: Pre-trained source-domain DNN          n: Number of layers in M_S
**Output:**
          M_DTL: Best trained DTL model
**Begin**
          best_score ← 0          M_DTL ← NULL          **for** i = 1 to n **do**                    Initialize DTL model M_T                    Transfer i layers from M_S to M_T                    Freeze the transferred layers                    Add target-specific trainable layers to M_T                    Train M_T using (x_t, y_t)                    Evaluate M_T using (x_tv, y_tv)                    Compute validation performance                    **if** validation score > best_score then                              best_score ← validation score                              M_DTL ← M_T                    **end if**          **end for**          Evaluate M_DTL on the unseen IoT-23 test set          Return M_DTL
**End**


## 6. Results and Discussion

### 6.1. Performance Evaluation

When evaluating models, it is helpful to look at a range of different evaluation metrics. We used the following evaluation metrics: accuracy (A), precision (P), recall (R), F1 value (F1), confusion matrix (CF) and area under the curve ROC score (AUC), model size, compression ratio (CR) and latency [13]. We also looked at training time taken per epoch for all the models.

Accuracy was used to evaluate the overall performance of DNNs and DTLs. Precision, recall, F1 value and confusion matrix were used to evaluate the performance of the classes.

Accuracy is the number of correct predictions divided by the total number of predictions. Accuracy is a good metric for data with balanced classes. It considers the true positive (TP), false positive (FP), true negative (TN) and false negative (FN).

A = (TP + TN)/(TP + FP + TN + FN)(1)

Precision is the number of true positive predictions divided by the total number of positive predictions (true and false) by the model.

P = TP/(TP + FP)(2)

Recall is the number of true positive predictions divided by all the positive instances.

R = TP/(TP + FN)(3)

Both precision and recall are scored between zero and one but scoring well on one may mean scoring poorly on the other.

F1 score is a metric that combines precision and recall. It determines how precise and robust a model is.

F1 = 2PR/(P + R)(4)

The area under the curve (AUC) score measures the quality of the model’s predictions. It is created by plotting the true positive rate (TPR) against the false positive rate (FPR). The space under the receiver operating characteristic (ROC) curve is represented as the AUC score. AUC counters the adverse effects of class imbalance.

An ROC curve is a graphical plot of the trade-off between the TPR and FPR, obtained by varying the classification threshold. The AUC-ROC captures the ROC trade-off as a comprehensive metric across all possible classification thresholds (i.e., the area under the ROC curve). The AUC-ROC metric is considered a comprehensive metric for comparison of binary classification models.

Compression ratio is a measure of how much a model has been reduced in size after applying compression techniques such and pruning and quantization.

CR = Original Model Size/Compressed Model Size(5)

**Figure 3:**
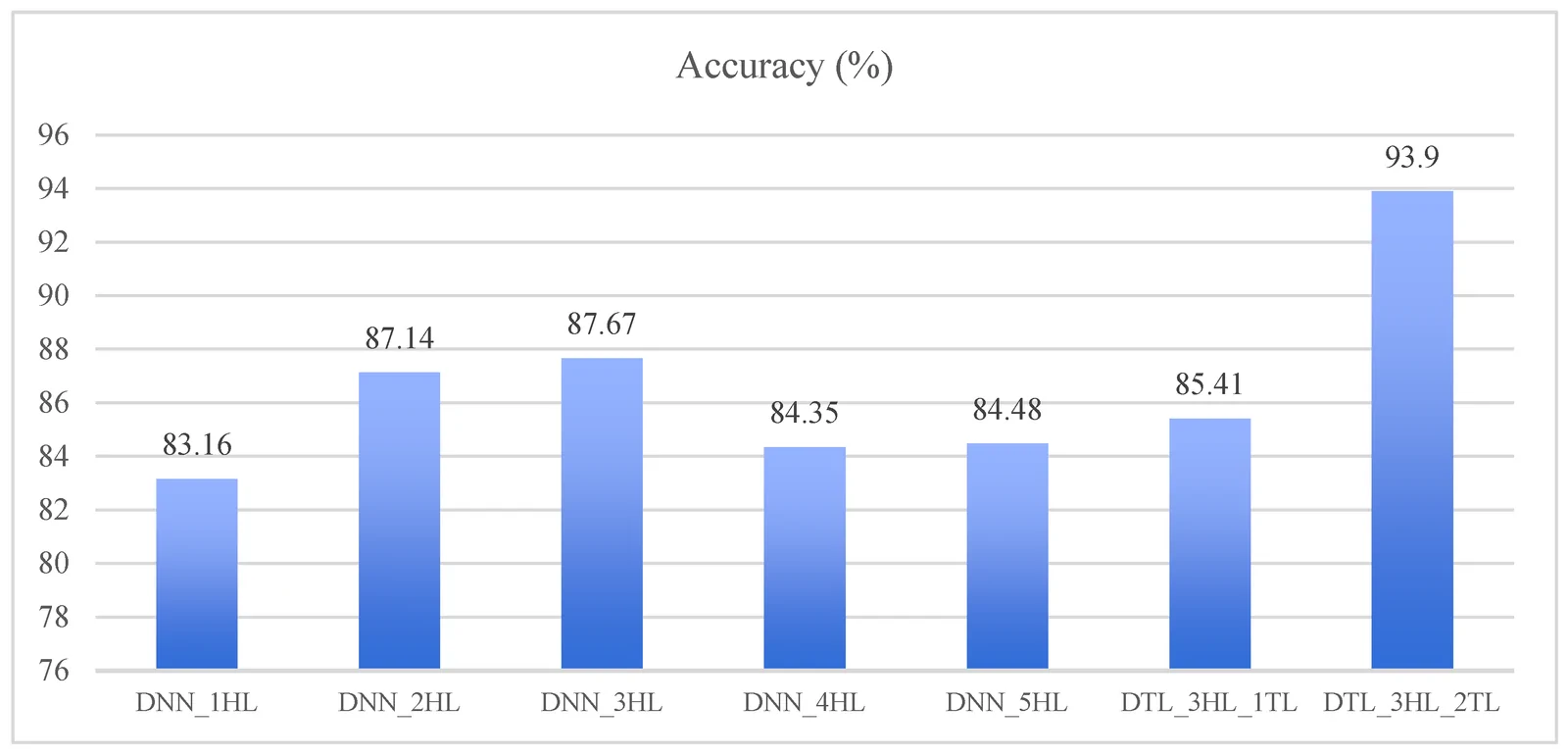
Accuracy comparison of models.

Accuracies of the models we experimented with are shown in Table 11 and Figure 3.

The table and figure clearly illustrate that the accuracy of a deep transfer learning model with three hidden layers where two layers are from the base model is higher than the other models. The accuracy of the deep transfer learning model was 93.9% compared to 87.67% of deep neural network trained on just the IoT data.

**Figure 4:**
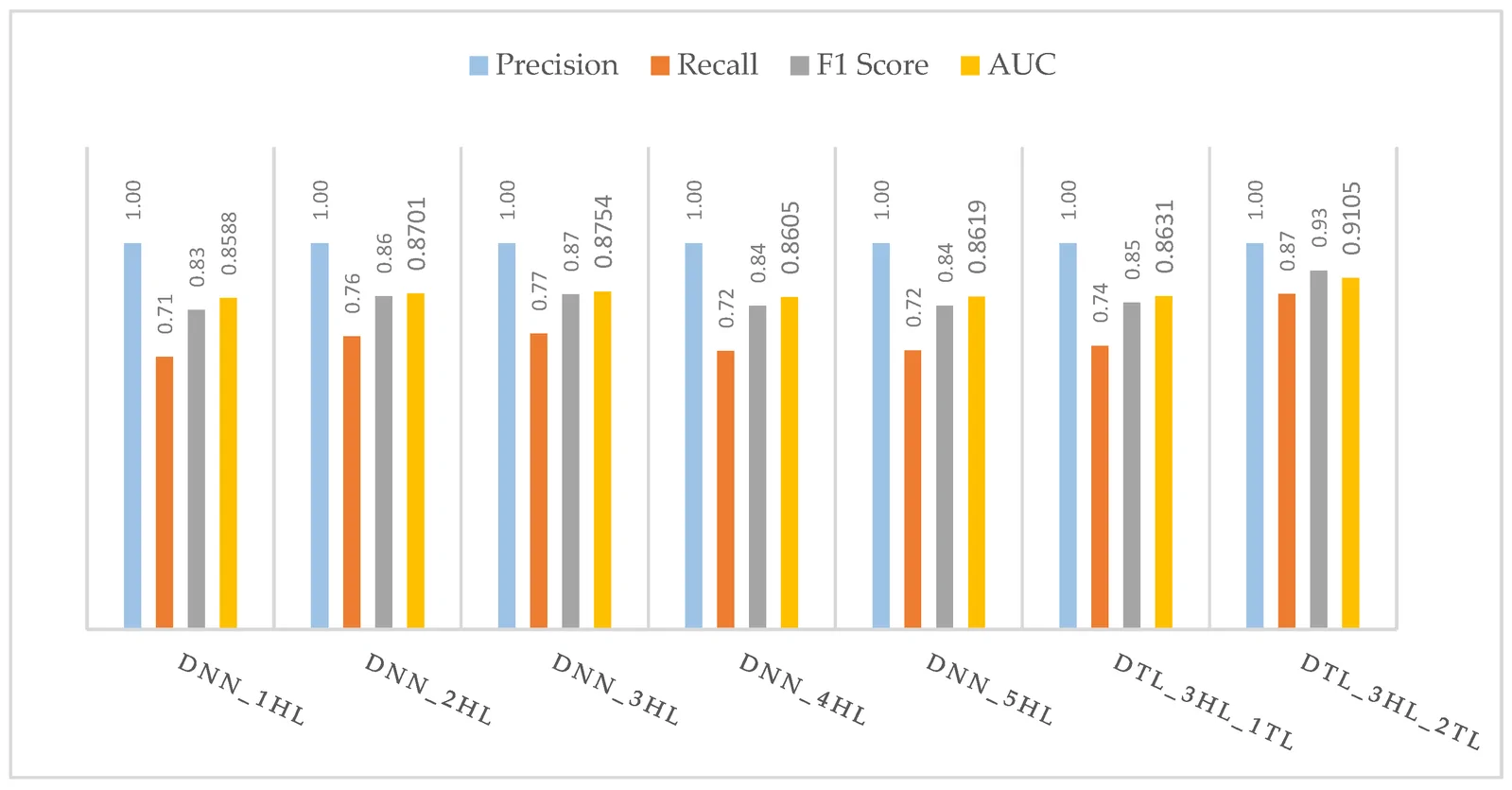
Comparison of precision, recall, F1 score, and AUC.

Comparison of precision, recall, F1 score and AUC is shown in Table 11 and Figure 4.

It can be noted that the precision is same in both models; however, the recall and F1 score are better for the deep transfer learning model which is 0.87 and 0.93 for DTL compared to 0.77 and 0.87 for DNN.

High precision models ensure that all predictions labeled positive are indeed positive. High recall models are likely to recall many of the true positive instances, at the cost of incurring many false positives.

**Figure 5:**
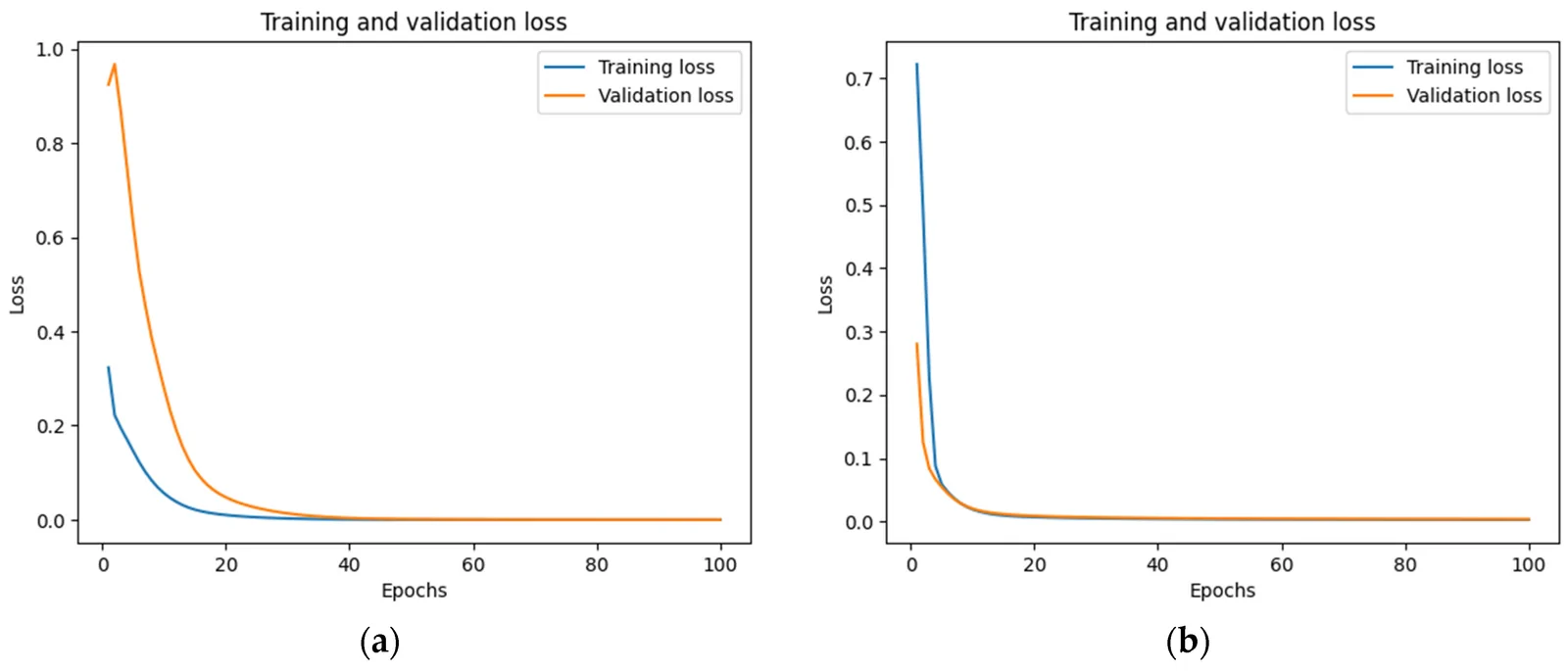
Loss curves. (**a**) Loss curve of DNN. (**b**) Loss curve of DTL.

The loss curves of DNN and DTL are shown in Figure 5. The loss curves indicate that the proposed DTL framework converges more rapidly than the baseline DNN model.

Furthermore, the DTL model achieves a lower validation loss, suggesting more effective feature learning. The reduced number of epochs required for convergence also demonstrates the efficiency of transferring knowledge from the source domain.

**Figure 6:**
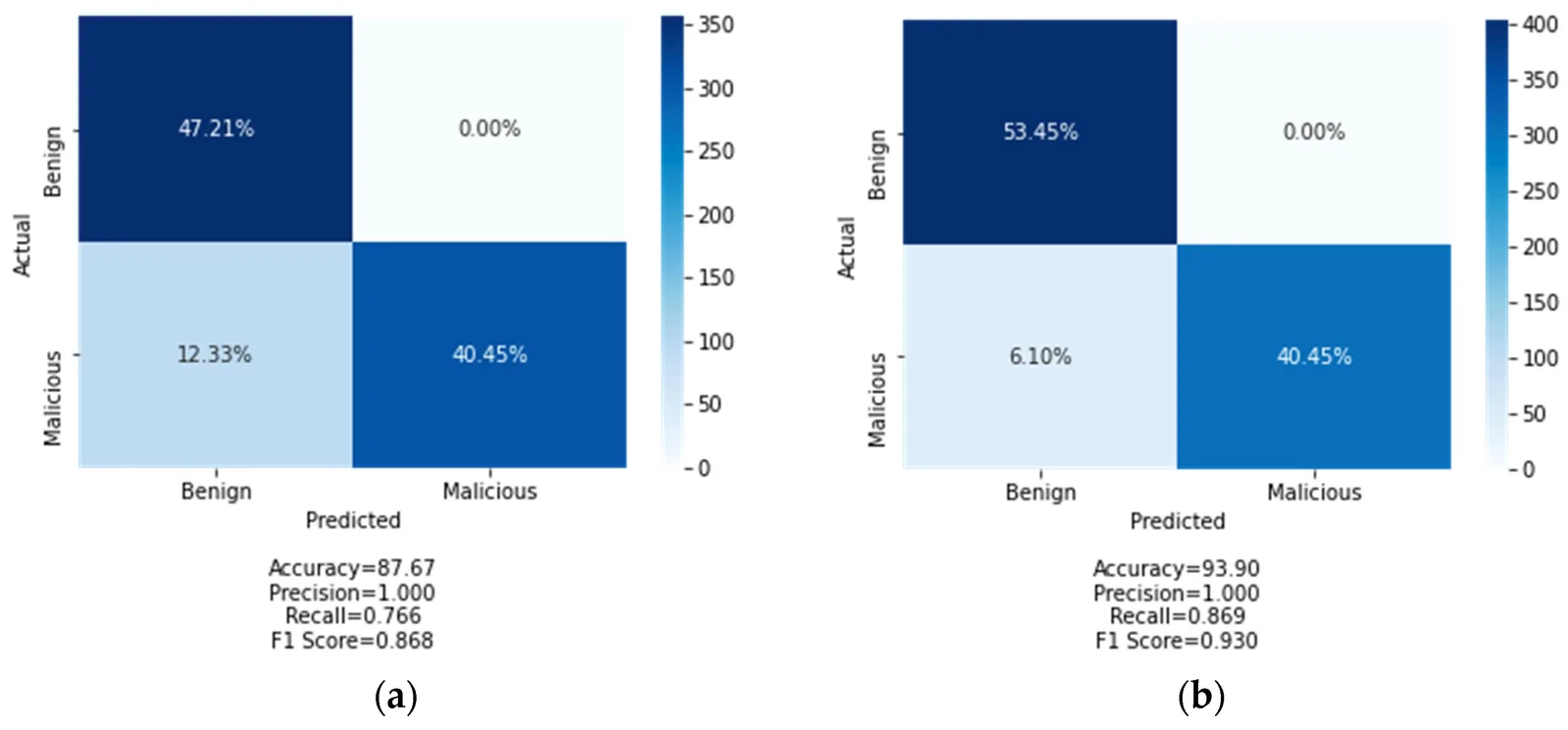
Confusion matrices. (**a**) Confusion matrix of DNN. (**b**) Confusion matrix of DTL.

The confusion matrices of DNN and DTL are shown in Figure 6. Intrusion detection in IoT encounters many challenges due to constrained resources and the complex environments the operate in.

Some common errors that can occur are

- False Positives: The system incorrectly identifies normal activity as malicious activity. This results in false alarms thereby expending precious resources and time in investigation benign activity.
- False Negatives: IDS fails to identify genuine malicious activity. This can lead to data breaches, unauthorized access, or harm to devices.
- Limited Detection Capabilities: IoT devices may have limited resources—processing power, memory, bandwidth—which can constrain the IDS’s capacity to effectively monitor and analyze traffic. This can result in shallow analysis that may permit sophisticated attacks to elude detection.

The cost of failing to detect an intrusion is very high. Therefore, we would prefer a model that makes sure the predictions labeled as benign have a high likelihood of being benign.

All the models that we experimented with achieved high precision. The DTL model correctly predicted 53.45% instances as benign compared to the DNN model which predicted 47% instances as benign. Both models predicted 40.45% instances as malicious. The DTL model incorrectly predicted 6.1% instances as benign when they were actually malicious, compared to the DNN which incorrectly predicted 12.33% instances. It can be noticed that the DTL model is better at identifying benign instances and is two times better at incorrect predictions.

Normalized percentages have been used to report results to enable consistent comparison of class-wise classification performance. Normalization expresses the proportion of benign and malicious samples correctly or incorrectly classified, making the confusion matrices less dependent on the absolute number of samples and facilitates comparison of false-positive and false-negative behavior between the DNN and DTL models.

**Figure 7:**
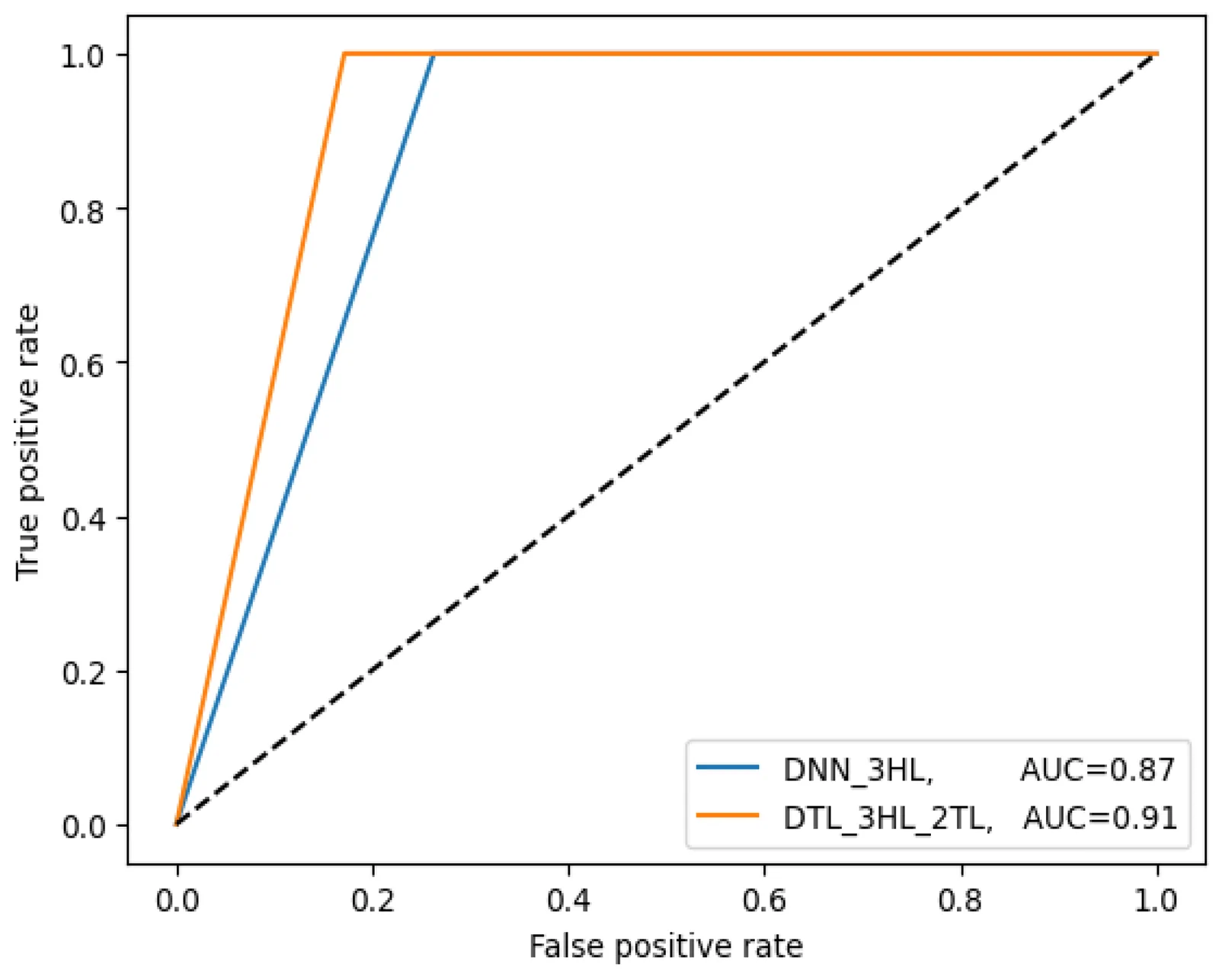
AUC-ROC of DNN and DTL.

Figure 7 shows the comparison of the AUC curves for DNN and DTL models. The DTL model has a higher AUC of 0.91 compared to the DNN models 0.87, indicating good performance in distinguishing between detecting benign and malicious network traffic.

**Figure 8:**
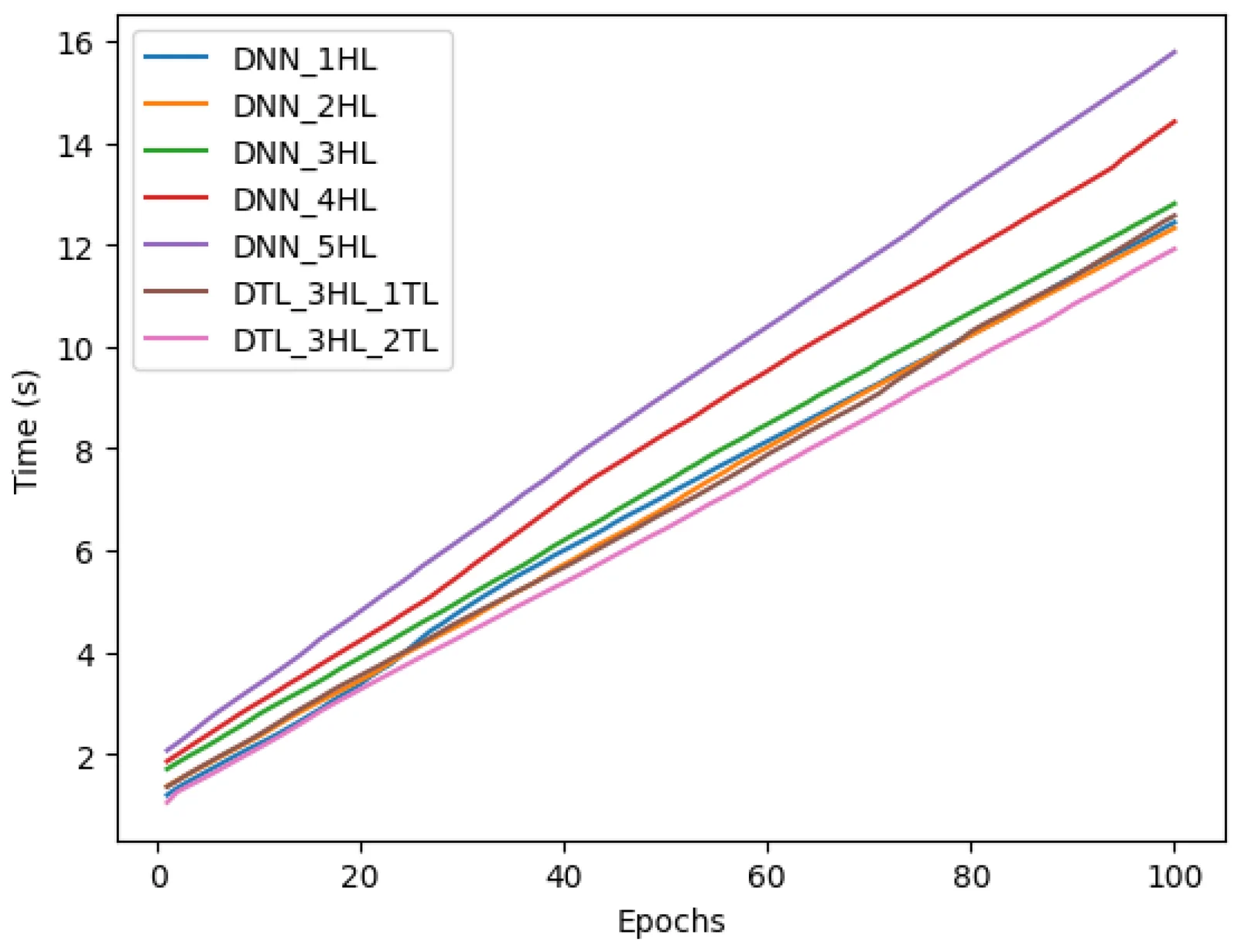
Time taken per epoch.

There are several factors that can affect the training time of a machine learning model including but not limited to datasets, model architecture, and hardware resources. Figure 8 shows the time taken in seconds by each model to complete 100 epochs. Clearly the final DTL model (DTL_2TL) outperforms all other models.

We also noticed that when knowledge from only one layer is transferred no performance improvement was noticed compared to a three hidden layer DNN.

Our results demonstrate that all evaluation metrics are better for a deep transfer learning model with three hidden layers where two layers are from the base model. It achieved the best accuracy, has the best recall, F1 score and AUC. This model also took less time to train compared to a three hidden layer DNN.

### 6.2. Ablation Study

To evaluate the contribution of the individual components of the proposed DTL framework, an ablation study was conducted, as summarized in Table 12. Specifically, the performance of the complete model was compared against several reduced variants in which key components were modified. The evaluated configurations included: (i) a baseline model trained solely on the target IoT dataset without transfer learning, (ii) a model utilizing source-domain data to measure source-domain usefulness, (iii) a transfer learning model utilizing some source-domain knowledge, and (iv) the full DTL framework incorporating cross-domain knowledge transfer and target-domain. This experimental design enables the impact of each component on intrusion detection performance to be isolated and quantified.

The results demonstrate that the full DTL framework consistently outperforms the reduced variants across the evaluated performance metrics. The baseline model exhibited the lowest performance due to the limited availability of training data, while the transfer learning variant achieved improved results by leveraging knowledge acquired from the source domain.

Adding the transfer learning component resulted in a 6.23% increase in accuracy. These findings confirm the effectiveness of the proposed framework and highlight the contribution of each component to improving performance in data-constrained IoT environments.

### 6.3. Evaluation Under Data-Constrained Conditions

A primary objective of the proposed framework is to improve intrusion detection performance in IoT environments where labeled training data are limited. Therefore, a data-constrained condition is defined as a scenario in which only a small proportion of the available labeled IoT data can be used to train the target-domain model. Such conditions commonly arise in practical IoT deployments due to the high cost of data annotation, privacy restrictions, the emergence of new devices, and the rarity of representative attack samples.

To evaluate the effectiveness of cross-domain transfer learning under realistic data scarcity, controlled experiments were conducted using progressively larger fractions of the labeled IoT-23 training data. Specifically, four target-domain training subsets containing 1%, 5%, 10%, and 25% of the available labeled training samples were constructed using stratified sampling to preserve the benign and malicious class distributions. The remaining labeled data were reserved for validation and testing to ensure fair comparison across all experiments.

For each target-data fraction, two models were trained and evaluated:

1. Target-Only DNN: A deep neural network initialized with random weights and trained exclusively on the selected IoT-23 subset.
2. Proposed DTL Model: A deep neural network pre-trained on the CIC-IDS2017 dataset and subsequently fine-tuned using the same IoT-23 subset.

All experiments employed identical network architectures, optimization parameters, batch sizes, stopping criteria, and evaluation metrics to isolate the effect of transfer learning. To improve the reliability and reproducibility of the results, each experiment was conducted using five different random seeds and the results were averaged across the five runs. Model performance was assessed using accuracy. The results of the experiments are presented in Table 13.

The performance gap between the transfer learning model and the target-only model is largest under the smallest training subsets, where labeled data are genuinely scarce. As the amount of target-domain data increases, the target-only model gradually approaches the performance of the transfer learning model because sufficient IoT-specific knowledge becomes available during training. These experiments provide direct evidence that the primary advantage of cross-domain transfer learning lies in reducing dependence on large quantities of labeled IoT intrusion data.

### 6.4. Transfer Layer Analysis

Because excessive parameter transfer may introduce domain-specific biases, an evaluation was conducted to determine the appropriate number of transferred layers. Different transfer configurations were evaluated by freezing varying numbers of pre-trained layers while retraining the remaining layers using IoT-23 data.

The results in Table 14 indicate that freezing the first two layers while fine-tuning the remaining layers provides the best balance between preserving generalized knowledge from the source domain and adapting to IoT-specific traffic characteristics. When only a single layer is transferred, the model does not fully exploit the knowledge learned from the source domain. These findings suggest that transferring low-level feature representations while allowing higher-level representations to adapt is an effective strategy for mitigating negative transfer.

### 6.5. Compression Analysis

Since the proposed framework targets resource-constrained IoT edge devices, additional experiments were conducted to evaluate the effectiveness of model compression techniques in reducing model size and inference latency while maintaining high intrusion detection performance. We have used a combination of two compression techniques, pruning and quantization. We first pruned the DTL model, then applied quantization on the pruned DTL model.

#### 6.5.1. Pruning

**Figure 9:**
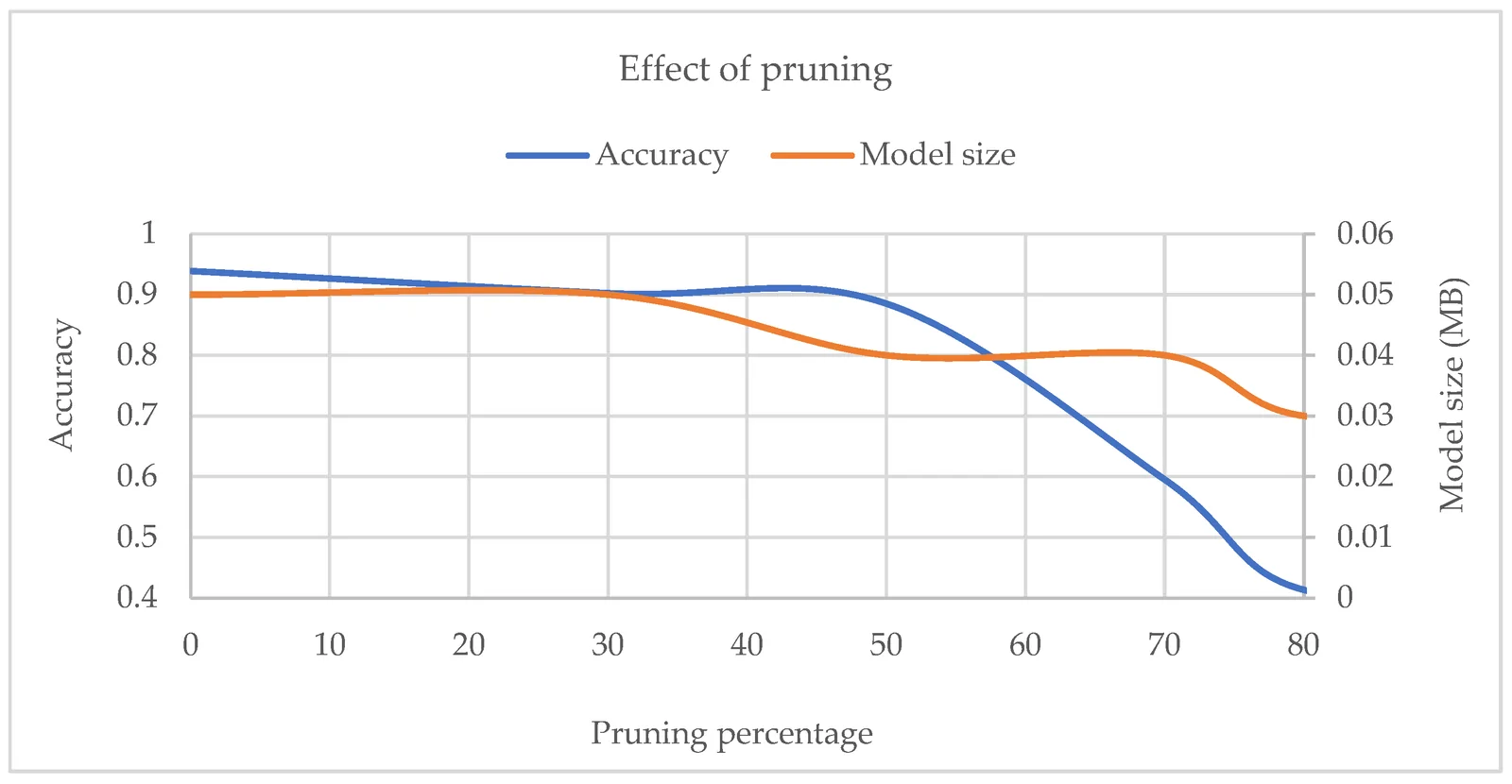
Effect of pruning.

For pruning we used the magnitude-based weight pruning technique, where four configurations were evaluated, corresponding to pruning levels of 0%, 30%, 50%, and 70%. The effect of pruning on model accuracy and model size is shown in Figure 9.

As can be noticed from the figure, at a pruning level of 30%, the model achieved performance metrics that were nearly identical to those of the uncompressed model, with only a negligible reduction in accuracy. However, there was no significant reduction in model size. Increasing the pruning level to 50% resulted in a slight reduction in accuracy (0.88) and model size (0.04 MB). The overall performance remained highly competitive. At the highest pruning level of 70%, a more pronounced decline in performance was observed. The accuracy fell to 0.59 while the model size remained at 0.04 MB. Reduction in accuracy indicates that excessive parameter removal begins to degrade the network’s ability to learn and represent complex attack patterns.

Among the evaluated configurations, the 50% pruning configuration provides a good balance between model accuracy and size.

#### 6.5.2. Quantization

In addition to 50% pruning, quantization was applied to further reduce the model size and optimize the proposed framework for deployment on resource-constrained IoT devices. A 50% pruning combined with the conversion of model parameters from 32-bit floating-point representations to 8-bit integer representations resulted in a substantial reduction in memory footprint and storage requirements. The experimental results indicate that the combination of pruning and quantization achieved a 5.9× reduction in model size; however, this is accompanied by a measurable decrease in detection accuracy. The accuracy decreases from 93.90% for the original DTL model to 88.32% after pruning and INT8 quantization, representing a reduction of 5.58 percentage points. This decrease indicates that the applied compression techniques introduce a measurable performance cost. Nevertheless, the compressed model provides substantial reductions in model complexity and resource requirements, which can facilitate deployment on resource-limited IoT edge devices. Table 15 presents the impact of the compression methods used on the performance and efficiency of the proposed framework. These characteristics are particularly important for intrusion detection applications deployed on IoT edge devices, where processing power, memory, and energy resources are often limited.

These findings indicate that quantization can complement pruning by providing additional reductions in model size and resource requirements. However, these gains must be considered alongside the associated impact on detection performance. Therefore, the degree of compression should be selected according to the requirements and constraints of the target deployment environment, balancing detection accuracy with computational cost and memory usage. Overall, the combination of pruning and quantization provides an approach for deploying the proposed deep transfer learning framework in resource-limited IoT environments.

### 6.6. Edge Deployment Configuration and Deployability

The proposed framework is designed for deployment in resource-constrained IoT edge environments where intrusion detection must be performed close to the data source while minimizing computational overhead and communication latency. To evaluate the feasibility of deployment, a representative edge-computing platform was considered consisting of an ARM-based embedded system comparable to widely used IoT gateways, such as a Raspberry Pi 5 or similar single-board computer. Such platforms typically incorporate a 64-bit quad-core ARM processor, 4–8 GB of memory, and a Linux-based operating system, providing a realistic execution environment for lightweight deep learning applications.

The compressed DTL model was developed with these hardware constraints in mind. Model pruning removes redundant network parameters, while post-training quantization reduces numerical precision from floating-point to integer representations, substantially decreasing the model size, memory footprint, and computational complexity. These optimizations enable the model to satisfy the limited storage, memory, and processing capabilities commonly found in IoT edge devices while preserving high intrusion detection performance.

The target deployment architecture consists of two functional components. First, network traffic is collected and preprocessed locally at the IoT gateway. Second, the compressed DTL model performs binary classification of network traffic as benign or malicious using TensorFlow Lite or an equivalent lightweight inference engine optimized for ARM processors.

Although comprehensive hardware benchmarking on multiple IoT platforms is beyond the scope of the present work, the proposed deployment architecture demonstrates that the compressed DTL model has resource requirements compatible with contemporary ARM-based edge devices. The combination of transfer learning, model compression, and lightweight inference makes the framework well suited for deployment in smart homes, industrial IoT, healthcare systems, and smart city infrastructures where security monitoring and efficient resource utilization are critical.

### 6.7. Discussion of Domain Differences and Limitations

Despite the encouraging experimental results, several limitations should be acknowledged. First, although CIC-IDS2017 and IoT-23 share common attack behaviors, they originate from different operational environments with distinct traffic characteristics, protocol distributions, and device types. This domain discrepancy introduces the possibility of negative transfer, where knowledge learned from the source domain may not fully generalize to IoT-specific traffic patterns. While the experimental evaluation demonstrates that the selected transfer strategy improves overall detection performance, its effectiveness ultimately depends on the degree of similarity between the source and target domains.

Second, the proposed framework performs binary classification and was evaluated using known attack categories represented in the training datasets. Consequently, its performance against previously unseen zero-day attacks or rapidly evolving adversarial behaviors may be limited. Although cross-domain transfer learning improves generalization compared with training exclusively on limited IoT data, the model remains dependent on the availability of representative malicious behaviors during pre-training.

Furthermore, IoT environments are inherently dynamic, with new devices, communication protocols, firmware versions, and attack techniques continuously emerging. Such changes may gradually alter the underlying data distribution, leading to concept drift and reduced detection performance over time. Addressing these challenges will require continual learning, domain adaptation, or periodic model retraining using newly collected network traffic.

## 7. Conclusions and Future Work

Intrusion detection is a critical component of IoT security. Deep learning techniques have demonstrated considerable success in intrusion detection tasks; however, their effectiveness often depends on the availability of large volumes of high-quality training data. In many IoT environments, the collection and labeling of representative datasets are challenging, resulting in data-constrained conditions that can limit model performance. To address this issue, this study investigated the effectiveness of deep transfer learning for IoT intrusion detection. Experimental results indicate that a deep transfer learning model trained using both computer network and IoT data achieves superior performance compared with a deep neural network trained solely on IoT data.

In the proposed framework, computer network intrusion data were used as the source domain to pre-train a three-layer deep neural network. During the transfer learning phase, the majority of the pre-trained model parameters were retained, while additional layers were introduced and fine-tuned using IoT intrusion data from the target domain. This cross-domain knowledge transfer enabled the model to leverage information learned from computer network traffic and adapt it to IoT intrusion detection tasks. The resulting framework achieved improvements in accuracy (93.9%), recall (0.87), F1-score (0.93), and AUC (0.91), while also reducing training time.

The incorporation of model compression further extends the applicability of the proposed framework to resource-constrained IoT edge environments. The combination of cross-domain deep transfer learning, pruning, and quantization enables the development of lightweight intrusion detection models that maintain strong predictive performance while significantly reducing memory overhead.

Consequently, the proposed framework demonstrates the potential of cross-domain knowledge transfer to address both the challenges of data scarcity and resource limitations, providing a practical and scalable solution for securing IoT systems operating in data-constrained environments.

In future work, we plan to extend our framework by exploring multiple source domains, including datasets from different IoT applications, alternative neural network architectures, and generative AI techniques. We also aim to extend the current binary classification task to multiclass classification, enabling the model to distinguish among different malware scenarios and attack families. In addition, we plan to incorporate continual learning capabilities to enable adaptation to emerging attack patterns and evolving network conditions, and to deploy the models on at least one representative edge-computing platform to further evaluate their practical resource requirements and deployment feasibility.

## Figures and Tables

**Table 1:** Comparison of related works and our work.

Ref.	Approach	Application	Addresses Data Scarcity	Cross-Domain Transfer	Model Compression	Edge Deployment	Key Features
[15]	Transfer Learning	Anomaly Detection	✓	✗	✗	✗	Anomaly detection in IoT traffic data
[16]	Transfer Learning	Intrusion Detection	✓	✗	✗	✗	Tailored for 5G IoT networks
[17]	Transfer Learning	Wastewater Treatment	✓	✗	✗	✗	Improves performance in industrial systems with limited data
[18]	Transfer Learning	Bearing Fault Diagnosis	✓	✗	✗	✗	Fault diagnosis under data scarcity
[19]	Transfer Learning	HVAC Forecasting	✓	✗	✗	✗	Attention mechanisms for forecasting
[20]	Federated Learning	IoT Model Training	✓	✗	✗	✓	Collaborative learning for resource-constrained IoT devices
[21]	Federated Transfer Learning	Sensor Fault Classification	✓	✗	✗	✓	Combines federated and transfer learning for sensor fault classification
[23]	Few-Shot Learning	Intrusion Detection	✓	✗	✗	✗	Detects unseen IoT attacks using limited labeled samples
[24]	Few-Shot Learning	Energy Forecasting	✓	✗	✗	✗	Forecasting of time-series data with scarce historical data
[25]	Meta-Learning	Intrusion Detection	✓	✗	✗	✗	Siamese-network-based intrusion detection with minimal labeled data
[26]	Meta + Federated Learning	Fault Diagnosis	✓	✗	✗	✓	Integrates meta-learning with federated learning
[27,28,29,30,31]	Synthetic Data Generation	Security and Prediction	✓	✗	✗	✗	Uses generative AI and GAN-based techniques to augment limited datasets
Our work	Deep Transfer Learning + Pruning + Quantization	Intrusion Detection	✓	✓	✓	✓	Transfers knowledge from computer network and IoT domains in data-constrained IoT edge environments

**Table 2:** Data distribution.

Dataset	Attack Type	Total
CIC-IDS2017	Benign Malicious Total	2,359,087 471,453 2,830,540
IoT-23	Benign Malicious Total	30,858,735 80,596,315 111,455,050

**Table 3:** Summary of CIC-IDS2017 and IoT-23 datasets.

Characteristic	CIC-IDS2017 (Source)	IoT-23 (Target)	Implications for Transfer Learning
Network Environment	Enterprise computer network	IoT devices and smart environments	Different operating environments require domain adaptation.
Traffic Characteristics	High-bandwidth TCP/IP communication	Lightweight, heterogeneous IoT communications	Low-level traffic features remain transferable despite protocol differences.
Device Types	Desktop computers, servers, workstations	Smart cameras, gateways, sensors, embedded devices	Device behavior differs, but attack behavior often exhibits common characteristics.
Primary Purpose	Enterprise intrusion detection	IoT intrusion detection	Supports cross-domain feature transfer.

**Table 4:** Capture assignments of IoT-23 dataset.

Partition	Benign	Malicious	Total	Source Captures
Training	2500	2500	5000	CTU-IoT-Malware-Capture-3-1, 8-1, 9-1, 17-1, 20-1, 34-1, 35-1, 36-1, 39-1, 42-1, 43-1, 49-1, 52-1, 60-1
Validation	500	500	1000	CTU-IoT-Malware-Capture-21-1, 33-1, 48-1
Testing	1000	1000	2000	CTU-Honeypot-Capture-7-1, CTU-IoT-Malware-Capture-1-1, 44-1

**Table 5:** Sampling strategy.

Dataset	Stage	Sampling Strategy	Purpose
CIC-IDS2017	Raw → cleaned	Remove invalid/duplicate records	Data quality
CIC-IDS2017	Cleaned → subset	Stratified by benign/malicious and attack category	Preserve source diversity
IoT-23	Raw → cleaned	Remove invalid/duplicate records	Data quality
IoT-23	Cleaned → split	Capture-disjoint	Prevent leakage
IoT-23	Training → subset	Capture-aware + benign/malicious stratification	Representative target data
IoT-23	Training subsets	1%, 5%, 10%, 25%	Data scarcity experiments
IoT-23	Test	Fixed; no sampling	Unbiased evaluation

**Table 6:** Original features.

Dataset	Original Features
CIC-IDS2017	Destination Port, Flow Duration, Total Fwd Packets, Total Backward Packets, Total Length of Fwd Packets, Total Length of Bwd Packets, Fwd Packet Length Max, Fwd Packet Length Min, Fwd Packet Length Mean, Fwd Packet Length Std, Bwd Packet Length Max, Bwd Packet Length Min, Bwd Packet Length Mean, Bwd Packet Length Std, Flow Bytes/s, Flow Packets/s, Flow IAT Mean, Flow IAT Std, Flow IAT Max, Flow IAT Min, Fwd IAT Total, Fwd IAT Mean, Fwd IAT Std, Fwd IAT Max, Fwd IAT Min, Bwd IAT Total, Bwd IAT Mean, Bwd IAT Std, Bwd IAT Max, Bwd IAT Min, Fwd PSH Flags, Bwd PSH Flags, Fwd URG Flags, Bwd URG Flags, Fwd Header Length, Bwd Header Length, Fwd Packets/s, Bwd Packets/s, Min Packet Length, Max Packet Length, Packet Length Mean, Packet Length Std, Packet Length Variance, FIN Flag Count, SYN Flag Count, RST Flag Count, PSH Flag Count, ACK Flag Count, URG Flag Count, CWE Flag Count, ECE Flag Count, Down/Up Ratio, Average Packet Size, Avg Fwd Segment Size, Avg Bwd Segment Size, Fwd Header Length, Fwd Avg Bytes/Bulk, Fwd Avg Packets/Bulk, Fwd Avg Bulk Rate, Bwd Avg Bytes/Bulk, Bwd Avg Packets/Bulk, Bwd Avg Bulk Rate, Subflow Fwd Packets, Subflow Fwd Bytes, Subflow Bwd Packets, Subflow Bwd Bytes, Init_Win_bytes_forward, Init_Win_bytes_backward, act_data_pkt_fwd, min_seg_size_forward, Active Mean, Active Std, Active Max, Active Min, Idle Mean, Idle Std, Idle Max, Idle Min
IoT-23	ts, uid, id.orig_h, id.orig_p, id.resp_h, id.resp_p, proto, service, duration, orig_bytes, resp_bytes, conn_state, local_orig, local_resp, missed_bytes, history, orig_pkts, orig_ip_bytes, resp_pkts, resp_ip_bytes, tunnel_parents

**Table 7:** Removed features.

Dataset	Removed Features
CIC-IDS2017	act_data_pkt_fwd, Active Max, Active Mean, Active Min, Active Std, Bwd Avg Bulk Rate, Bwd Avg Bytes/Bulk, Bwd Avg Packets/Bulk, Bwd Header Length, Bwd IAT Max, Bwd IAT Mean, Bwd IAT Min, Bwd IAT Std, Bwd IAT Total, Bwd Packet Length Max, Bwd Packet Length Mean, Bwd Packet Length Min, Bwd Packet Length Std, Bwd PSH Flags, Bwd URG Flags, CWE Flag Count, ECE Flag Count, Flow IAT Max, Flow IAT Mean, Flow IAT Min, Flow IAT Std, Fwd Avg Bulk Rate, Fwd Avg Bytes/Bulk, Fwd Avg Packets/Bulk, Fwd IAT Max, Fwd IAT Mean, Fwd IAT Min, Fwd IAT Std, Fwd IAT Total, Fwd Packet Length Max, Fwd Packet Length Mean, Fwd Packet Length Min, Fwd Packet Length Std, Fwd PSH Flags, Fwd URG Flags, Idle Max, Idle Mean, Idle Min, Idle Std, Init_Win_bytes_backward, Init_Win_bytes_forward, Max Packet Length, Min Packet Length, min_seg_size_forward, Packet Length Mean, Packet Length Std, Packet Length Variance, PSH Flag Count, Subflow Bwd Packets, Subflow Fwd Packets, URG Flag Count
IoT-23	ts, uid, conn_state, detailed-label, id.orig_h, id.resp_h, id.orig_p, proto, service, local_orig, local_resp, missed_bytes, tunnel_parents

**Table 8:** Final feature list.

Feature	CIC-IDS2017	IoT-23	Alignment
Destination Port	Destination Port	id.resp_p	Direct semantic mapping
Flow Duration	Flow Duration	duration	Direct mapping
Forward/Origin Packets	Total Fwd Packets	orig_pkts	Direct semantic mapping
Backward/Response Packets	Total Backward Packets	resp_pkts	Direct semantic mapping
Forward/Origin Bytes	Total Length of Fwd Packets	orig_bytes	Approximate semantic mapping
Backward/Response Bytes	Total Length of Bwd Packets	resp_bytes	Approximate semantic mapping
Forward Packet Rate	Fwd Packets/s	orig_pkts/duration	Derived
Backward Packet Rate	Bwd Packets/s	resp_pkts/duration	Derived
Total Packet Rate	Flow Packets/s	(orig_pkts + resp_pkts)/duration	Derived
Total Byte Rate	Flow Bytes/s	(orig_bytes + resp_bytes)/duration	Derived
Average Packet Size	Average Packet Size	(orig_bytes + resp_bytes)/(orig_pkts + resp_pkts)	Derived
Forward Average Packet Size	Avg Fwd Segment Size	orig_bytes/orig_pkts	Derived
Backward Average Packet Size	Avg Bwd Segment Size	resp_bytes/resp_pkts	Derived
Down/Up Ratio	Down/Up Ratio	resp_bytes/orig_bytes	Derived
Total Byte Count	Fwd Bytes + Bwd Bytes	orig_bytes + resp_bytes	Derived
Total Packet Count	Fwd Packets + Bwd Packets	orig_pkts + resp_pkts	Derived
Forward Byte Ratio	Fwd bytes/total bytes	orig_bytes/(orig_bytes + resp_bytes)	Derived
Backward Byte Ratio	Bwd bytes/total bytes	resp_bytes/(orig_bytes + resp_bytes)	Derived
Forward Packet Ratio	Fwd packets/total packets	orig_pkts/(orig_pkts + resp_pkts)	Derived
Backward Packet Ratio	Bwd packets/total packets	resp_pkts/(orig_pkts + resp_pkts)	Derived
Subflow Forward Bytes	Subflow Fwd Bytes	orig_ip_bytes	Approximate semantic mapping
Subflow Backward Bytes	Subflow Bwd Bytes	resp_ip_bytes	Approximate semantic mapping
FIN Flag Count	FIN Flag Count	history_ShAFf	Derived
SYN Flag Count	SYN Flag Count	history_S	Derived
RST Flag Count	RST Flag Count	history_ShADadfr	Derived
ACK Flag Count	ACK Flag Count	history_ShAFaf	Derived
Forward Backward Byte Ratio	Total Length of Fwd Packets/Total Length of Bwd Packets	orig_ip_bytes/resp_ip_bytes	Derived
Log Duration	log(Duration)	log(duration)	Transformation
Total Packets	Total Fwd Packets + Total Backward Packets	orig_pkts + resp_pkts	Derived

**Table 9:** DNN architecture.

Hyperparameter	Value
Hidden layers	3
Layer1 nodes	64
Layer2 nodes	32
Layer3 nodes	16
Activation (hidden layers)	ReLU
Regularizer	L2
L2 coefficient	0.001
Activation (output layer)	Sigmoid
Loss function	Binary cross-entropy
Optimizer	RMSProp
Learning rate	0.001
Metrics	Accuracy

**Table 10:** DTL architecture.

Hyperparameter	Value
Hidden layers	3
Layer1 nodes	64
Layer2 nodes	32
Layer3 nodes	16
Transfer layers	2
Activation (hidden layers)	ReLU
Regularizer	L2
L2 coefficient	0.001
Activation (output layer)	Sigmoid
Loss function	Binary cross-entropy
Optimizer	RMSProp
Learning rate	0.001
Metrics	Accuracy

**Table 11:** Metrics of models.

Model	Accuracy (%)	Precision	Recall	F1 Score	AUC (%)
DNN_1HL	83.16	1.00	0.71	0.83	85.88
DNN_2HL	87.14	1.00	0.76	0.86	87.01
DNN_3HL	87.67	1.00	0.77	0.87	87.54
DNN_4HL	84.35	1.00	0.72	0.84	86.05
DNN_5HL	84.48	1.00	0.72	0.84	86.19
DTL_3HL_1TL	85.41	1.00	0.74	0.85	86.31
DTL_3HL_2TL	93.90	1.00	0.87	0.93	91.05

**Table 12:** Ablation analysis of different configurations.

Configuration	Accuracy (%)
Target-only DNN	87.67
Source-only DNN	86.77
DTL	93.90

**Table 13:** Cross-domain transfer learning performance.

Training Subset	DNN Accuracy (%)	DTL Accuracy (%)	Improvement
1%	59.55 ± 0.33 (95% CI: 59.15–59.96)	81.56 ± 0.52 (95% CI: 80.91–82.21)	+22.01
5%	79.97 ± 3.60 (95% CI: 75.50–84.44)	84.08 ± 3.25 (95% CI: 80.04–88.12)	+4.11
10%	82.76 ± 2.85 (95% CI: 79.23–86.30)	86.21 ± 1.17 (95% CI: 84.76–87.66)	+3.45
25%	85.01 ± 1.13 (95% CI: 83.61–86.42)	87.43 ± 2.44 (95% CI: 84.40–90.46)	+2.42

**Table 14:** **Performance comparison of different** transfer layer configurations.

Model	Frozen Layers	Fine-Tuned Layers	Accuracy (%)	F1 Score	AUC (%)
Baseline DNN	0	All	87.67	0.87	87.54
DTL_1TL	1	2	85.41	0.85	86.31
DTL_2TL	2	1	93.90	0.93	91.05

**Table 15:** Metrics of models after compression.

Model	Accuracy (%)	F1	AUC (%)	Model Size (MB)	Compression Ratio	Latency (ms)
DTL	93.90	0.93	91.05	0.05	1.0	64.39
DTL + Pruning (50%)	88.54	0.87	89.24	0.04	1.3	60.11
DTL + Pruning (50%) + Quantization (INT8)	88.32	0.88	90.20	0.01	5.9	58.94

## Data Availability

The original contributions presented in this study are included in the article. Further inquiries can be directed to the corresponding author.
